# Kyste du ligament rond du foie mimant un kyste ovarien malin

**DOI:** 10.11604/pamj.2013.14.54.2239

**Published:** 2013-02-09

**Authors:** Sofia Jayi, My Abdelilah Melhouf

**Affiliations:** 1 Service de gynéco-obstétrique 2, CHU HASSAN II, Université Sidi Mohammed Benabdellah, Fès, Maroc

**Keywords:** Kyste, ligament rond, foie, kyste ovarien, cyst, round ligament, liver, ovarian cyst

## Image en médicine

**Figure 1:**
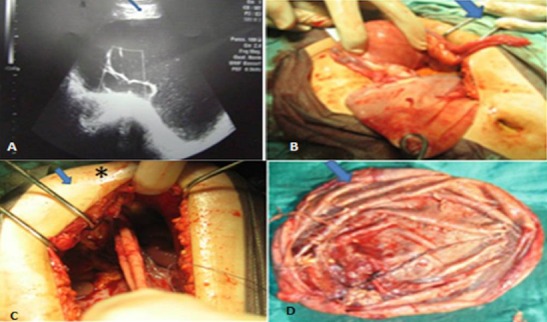
Kyste du ligament rond du foie mimant un kyste ovarien malin: trouvailles échographiques, résection du kyste au niveau de son collet et examen macroscopique de la pièce.

Mme A.M âgée de 29 ans multipare avec antécédent de cholécystotomie, a consulté pour douleur pelvienne chronique sans autre signes gynécologiques, digestifs ou urinaires chez qui l'examen trouve une masse abdomino- pelvienne arrivant jusqu’à l'ombilic rénitente sensible. Au toucher vaginal complété par le toucher rectale, la taille de l'utérus est difficile à apprécier avec perception du pole inférieur de la masse. l’échographie pelvienne (Figure 1-A) objective une image kystique dépassant l’écran, à contenu finement échogène, comportant une cloison incomplète épaisse (17mm) non vascularisée, l'utérus sans anomalie et les ovaires non pas été visualisés, par ailleurs pas d’épanchement intra-peritoneal, l'IRM pelvienne n'a pas été réalisée par manque de moyens. Ainsi une laparotomie exploratrice a été réalisée en ayant évoqué l'origine ovarienne maligne en premier lieu, cependant l'exploration a trouvé un kyste énorme appendu au ligament rond du foie, tandis que l'appareil génital interne était macroscopiquement intègre. Une résection du kyste au niveau de son collet - en collaboration avec les viscéralistes-a été réalisée après aspiration de son contenu qui été légèrement hématique (Figure 1-B, C). L'examen macroscopique de la pièce a trouvé de multiples fines cloisons incomplètes sur la paroi interne du kyste (Figure 1-D). Les suites opératoires étaient simples. L'examen anatomopathologique est revenu en faveur d'un kyste bénin du ligament rond du foie.

